# The relationship between perceptions and self-paid hepatitis B vaccination: A structural equation modeling approach

**DOI:** 10.1371/journal.pone.0208402

**Published:** 2018-12-06

**Authors:** Yogambigai Rajamoorthy, Alias Radam, Niazlin Mohd Taib, Khalid Ab Rahim, Abram Luther Wagner, Mudatsir Mudatsir, Subramaniam Munusamy, Harapan Harapan

**Affiliations:** 1 Department of Economics, Faculty of Accountancy and Management, Universiti Tunku Abdul Rahman, Kajang, Selangor, Malaysia; 2 Department of Economics, Faculty of Economics and Management, Universiti Putra Malaysia, Serdang, Selangor, Malaysia; 3 Department of Medical Microbiology and Parasitology, Faculty of Medicine and Health Sciences, Universiti Putra Malaysia, Serdang, Selangor, Malaysia; 4 Department of Epidemiology, University of Michigan, Ann Arbor, Michigan, United States of America; 5 Medical Research Unit, School of Medicine, Syiah Kuala University, Banda Aceh, Indonesia; 6 Department of Microbiology, School of Medicine, Syiah Kuala University, Banda Aceh, Indonesia; 7 School of Management and Business, Manipal International University, Nilai, Negeri Sembilan, Malaysia; 8 Tropical Disease Centre, School of Medicine, Syiah Kuala University, Banda Aceh, Indonesia; 9 School of Biomedical Sciences, University of Western Australia, Nedlands, Western Australia, Australia; Children's Mercy Hospitals and Clinics Department of Pathology and Laboratory Medicine, UNITED STATES

## Abstract

**Background:**

Malaysia has a comprehensive, publicly-funded immunization program for hepatitis B (HepB) among infants, but adults must pay for the vaccine. The number of HepB carriers among adults is expected to increase in the future; therefore, we examined the impact of five constructs (cues to action, perceived barriers, perceived benefit, perceived severity, and perceived susceptibility) on adults’ willingness to pay (WTP) for HepB vaccine; secondarily, we examined the association between perceived barriers and perceived benefits.

**Methods:**

Adults were selected through a stratified, two-stage cluster community sample in Selangor, Malaysia. The reliability, convergent validity, and discriminant validity of the measurement model were assessed before implementing a partial least squares structural equation model (PLS-SEM) to evaluate the significance of the structural paths.

**Results:**

A total of 728 participants were enrolled. The five constructs all showed adequate internal reliability, convergent validity, and discriminant validity. There was a significant, positive relationship to WTP from constructs (perceived barriers [Path coefficient (β) = 0.082, *P* = 0.036], perceived susceptibility [β = 0.214, *P*<0.001], and cues to action [β = 0.166, *P*<0.001]), and the model all together accounted for 8.8% of the variation in WTP. There was a significant, negative relationship between perceived barriers and perceived benefit [β = -0.261, *P*<0.001], which accounted for 6.8% of variation in perceived benefit.

**Conclusions:**

Policy and programs should be targeted that can modify individuals’ thoughts about disease risk, their obstacles in obtaining the preventive action, and their readiness to obtain a vaccine. Such programs include educational materials about disease risk and clinic visits that can pair HepB screening and vaccination.

## Introduction

Hepatitis B (HepB), caused by Hepatitis B virus (HBV), is a major public health problem in Malaysia [1, 2]. Its extensive impact on the health care system in the country arises from the large number of cases, a high reported incidence rate, and an increasing number of deaths, all of which are among the highest of any vaccine preventable disease [1–7]. The high seroprevalence of HBV surface antigen (HBsAg) in the general population (3–5%) and among chronic hepatitis patients (75.3%) highlights the burden of disease in the population and suggests that comprehensive preventive measures are warranted [4]. Greater knowledge about the uptake of HepB vaccine has become especially necessary given the World Health Organization (WHO)’s goal to eliminate HepB by 2030 [8].

Malaysia introduced free HepB vaccination for infants in 1989 and so the infection rate is expected to continually decrease in the future; however, adults are excluded from this programme (although vaccines are available to adults for a fee). In 2009, it was predicted that an inability to control HepB in adults will cause the number of HepB cases to rise in the foreseeable future [9]. Data from the Malaysian Ministry of Health revealed that the incidence of HepB has increased from 2.26 per 100,000 population in 2010 to 12.94 per 100,000 population in 2014 [5, 7]. One modeling study has predicted that the HepB incidence rate in Malaysia will continue to increase with an incidence rate of 122 per 100,000 population by 2030 [10]. Most of the previous HepB studies in Malaysia have prioritized analysing the disease as an occupational hazard. The medical occupation field in Malaysia requires all healthcare workers to receive HepB vaccination due to potential exposure to the HBV. Most health care workers have been found to be aware of the risk of HepB [11]; however, mandatory HepB vaccination was not well received by healthcare workers [11–13]. A study published in 1987 (when a plasma-derived vaccine was available) [12] and 2001 (when the current generation of vaccine was available) [13] found that the percentage of dental practitioners who had been vaccinated was 32% and 44%, respectively. Although there is a continuing increase of HepB vaccination coverage overtime, the latest study published in 2005 revealed that only 58.4% of healthcare workers had completed the HepB vaccine series [11]. These studies have indicated a complex interrelationship between knowledge of HepB, attitudes towards the HepB vaccine, and perceptions of the importance of receiving the vaccine.

Although studies related to HepB vaccination among healthcare workers are available in Malaysia [11–13], knowledge about perceptions of HepB and vaccination behaviour is lacking among the general populace [14]. Therefore, we undertook a study of perceptions of HepB and HepB vaccination among adults in the general population of Selangor, Malaysia, to determine the relationship between perceptions and willingness to pay (WTP) for HepB vaccination. The relationship of the variables were based on Health Belief Model (HBM) [15]. The Theory of Planned Behaviour (TPB) and HBM are two powerful psychological theories, which are commonly used to understand beliefs, values and attitudes related to a health behavior [16]. According to the HBM, health-related actions are informed by an individual’s (1) perceived susceptible to a health condition, (2) perceived severity of the health condition, (3) beliefs that taking a particular action would have a benefit, and (4) ability to overcome barriers [17]. The HBM is different from other models like the TPB, because there are no strict guidelines as to how the different variables predict behavior. HBM’s flexibility makes it highly adaptable and it can be used for a wide range of health issues [18–21]. In this present study, the constructs included cues to action, perceived barriers, perceived benefit, perceived severity, and perceived susceptibility. We also examined the association between perceived barriers and perceived benefits.

## Materials and methods

### Ethical statement

The study protocol was approved by the Institutional Review Board of Universiti Putra Malaysia, Selangor, Malaysia and all participants were anonymized. The aims, risks, and benefits of the study were explained to each participant, and they were asked to sign a consent form prior to enrolment in the study. Participants were also informed that they could quit at any time during the interview session. The work was carried out in accordance with The Code of Ethics of the World Medical Association (Declaration of Helsinki) for experiments involving humans.

### Study design and study sample

**Fig 1:**
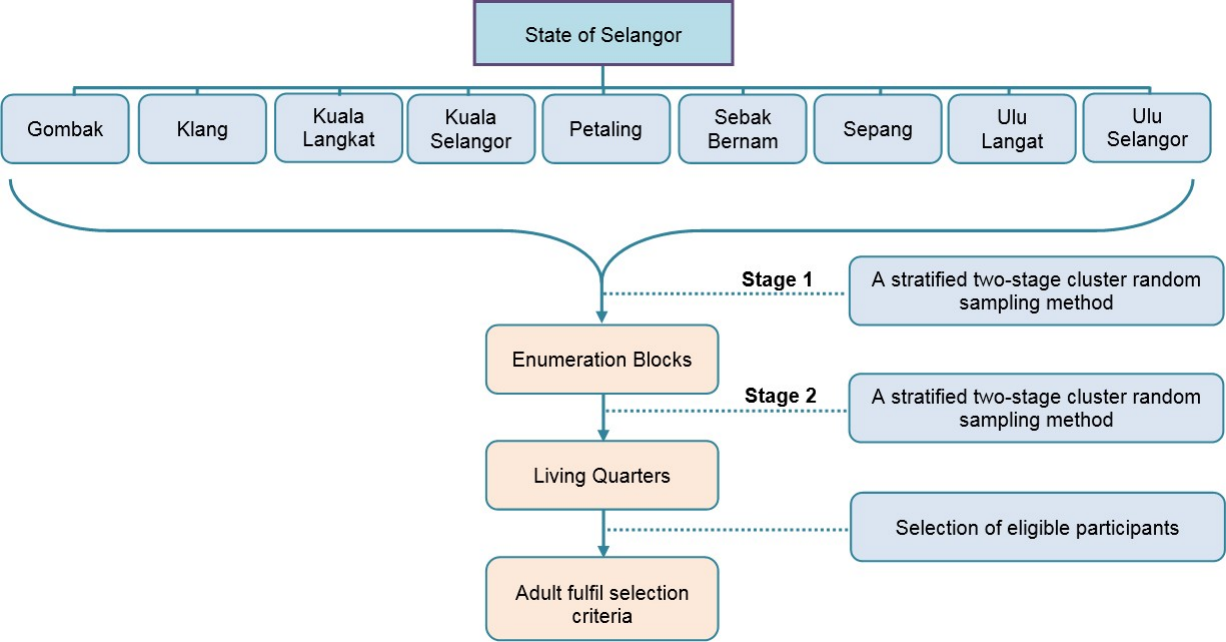
Flow chart of sampling methods used in the study.

This study was a part of a Malaysian Hepatitis B Project that have been described elsewhere [22]. A household cross-sectional survey was conducted in nine districts of Selangor, the most populous state in Malaysia with over 6.3 million inhabitants. The actual survey was carried out from January to May 2016. The sampling frame for this study was obtained from Department of Statistics, Malaysia (DOS) based on the National Population and Housing Census 2010. The maps with enumeration blocks of households in nine districts were purchased and a stratified two-stage cluster sampling design with proportional allocation was employed. The 1,575,200 households in Selangor were divided into 16,562 enumeration blocks in which each enumeration block consisted of 80–120 living quarters. Sixty-four out of 16,562 enumeration blocks were randomly selected and within each enumeration block, 12 living quarters were randomly selected (Fig 1). From each selected living quarter, one adult aged ≥20 years who was a Malaysian citizen was selected randomly. Face-to-face interviews were conducted to collect the data.

### Study instrument

**Fig 2:**
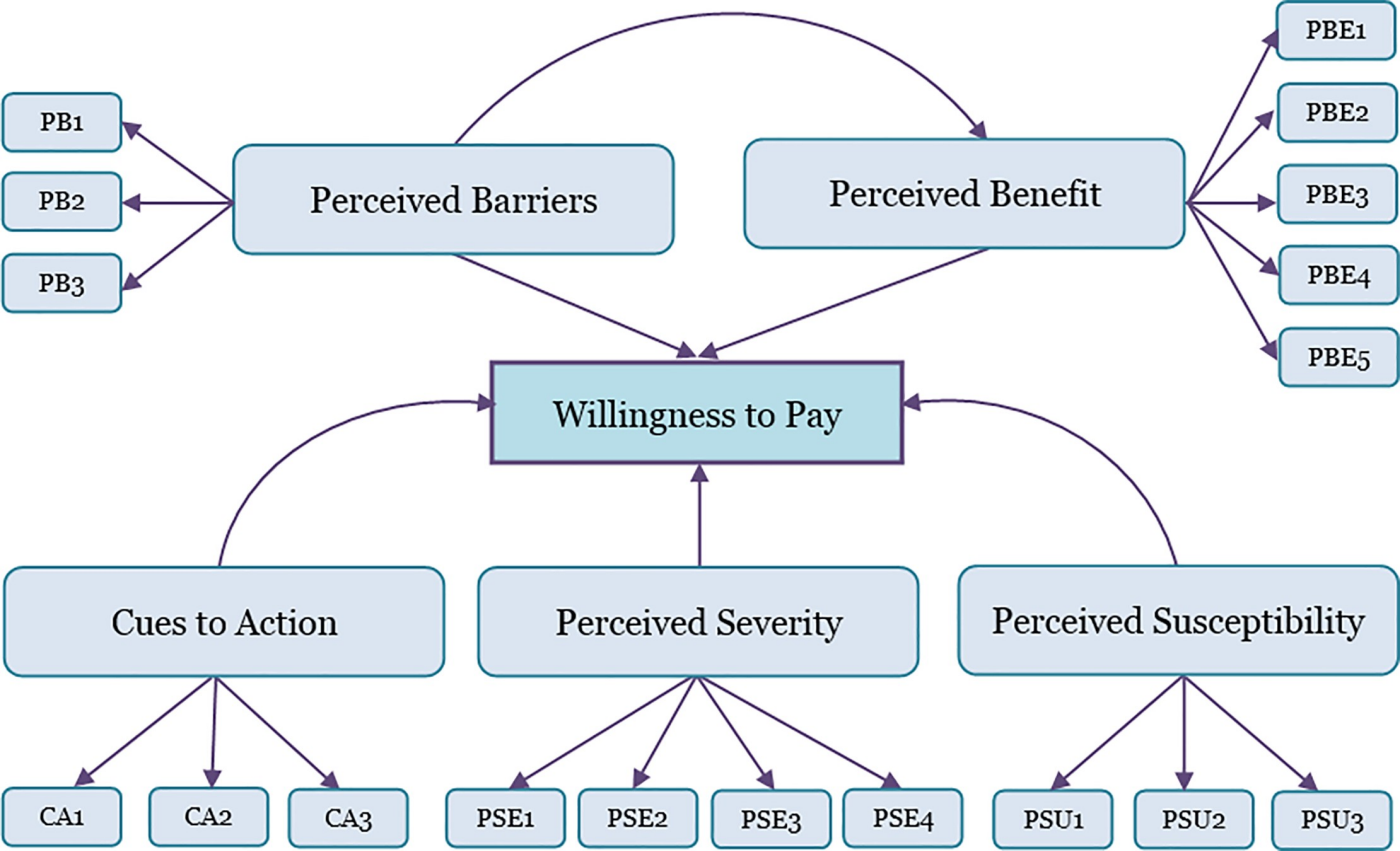
Theoretical framework and structural model of Partial Least Squares Structural Equation Modelling (PLS-SEM) in this study. The model shows hypothesized associations between cues to action, perceived barriers, perceived benefit, perceived severity and perceived susceptibility, and willingness to pay for hepatitis B vaccine.

The theoretical framework and structural model of Partial Least Squares Structural Equation Modelling (PLS-SEM) in this study is shown in Fig 2. The conceptual basis for this study was the Health Belief Model. The number of questions for each construct domain as follows: cues to action (3 items), perceived barriers to vaccination (3 items), perceived benefits of HepB vaccination (5 items), perceived severity of HepB disease (4 items), and perceived susceptibility to HepB infection (3 items) (Table 1). Participants’ WTP for HepB vaccine was assessed using a single question: “*I agree to pay for hepatitis B vaccine at the expense of my own*”. Each item within the perception constructs and WTP was measured on a seven-point Likert scale, ranging from 1 (strongly disagree) to 7 (strongly agree). The English version of this questionnaire was developed based on previous literature [18, 23–25] (Table 1) and translated into the Malay language. The detailed questionnaire used in this study is given in S1 Supporting Information file. Content validity of the English and Malay questionnaires was assessed using a scientific panel consisting of a medical microbiologist, a public health doctor and a nephrologist. A pilot study of 121 respondents, selected via a convenience sample in a public place was conducted to test the finalised questionnaire.

**Table 1:** Questionnaire items and their sources.

Constructs	Question	Adapted from
Cues to action		
CA1	I think the screening for HBV infection is a good practice	[18]
CA2	An additional dose (booster) of the vaccine for HepB should be taken when needed	[18]
CA3	I think all members of the family and friends should get the HepB vaccine	[18]
Perceived barriers		
PB1	I believe that the vaccination is not effective for me	[26]
PB2	I believe that the HepB vaccination is likely to cause more harm than good	[18]
PB3	I do not have the time to get the vaccination	[18]
Perceived benefit		
PBE1	I believe if I get the HepB vaccine, I shall be protected from HBV infection	[18]
PBE2	If I take the HepB vaccine, it will reduce my worry about liver disease	[18]
PBE3	I believe in the effectiveness of the HepB vaccine now	[25]
PBE4	I believe a vaccine for HepB strengthens the immune system against HBV	[18]
PBE5	I believe that getting the HepB vaccine is a good way to protect yourself from HBV infection	[25]
Perceived severity		
PSE1	I believe that I am at a higher risk of HBV infection	[18]
PSE2	I believe that my ethnic group is at a higher risk of HBV infection	[18]
PSE3	I believe that HBV infection is a serious disease	[18]
PSE4	I believe that HBV infection leads to death	[24]
Perceived susceptibility		
PSU1	I am less likely than most people to get infected with HepB	[23]
PSU2	My body could fight off HBV infection.	[23]
PSU3	I never worry about getting infected with HepB	[18]

### Statistical analysis

Descriptive statistics were computed for the demographic composition of the sample, along with the frequency of responses for all items of the constructs (i.e., cues to action, perceived barriers, perceived benefit, perceived severity and perceived susceptibility).

The study analysed the collected questionnaires using the partial least squares structural equation modelling (PLS-SEM) approach. In the context of this study, PLS-SEM was appropriate because (1) PLS-SEM does not required data to be normally distributed, (2) our sample size was large enough to establish a consistent PLS-SEM estimator and to increase precision of the results, (3) PLS-SEM handles single item (e.g., WTP) and multi-item (e.g., the perceptions and cues to action) constructs, and (4) PLS-SEM can be used for theory development; in this study, we used PLS-SEM to determine the relationship between perceived barriers and perceived benefits, two constructs whose association is that are less often assessed in studies using the Health Belief Model [27].

Before assessing the structural model of the constructs using PLS-SEM, based on the conceptual framework in Fig 1, multiple measures of reliability and validity of items within constructs were computed. The reliability of items within a construct was assessed using Crohnbach’s α and composite reliability. A Crohnbach’s α>0.7 and a composite reliability lower bound of 0.6 were considered acceptable [28, 29] and therefore were employed in this study. In addition, composite reliability values of more than 0.70 were used to judge a reliability as satisfactory [30]. Convergent validity, the extent to which a measure correlates positively with alternatives measures of the same items of the constructs, was examined using Average Variance Extracted (AVE) factor loadings. The AVE is measure of the amount of variance that is captured by a construct in relation to the amount of variance due to measurement error [31] and calculated as the grand mean value of the squared loadings of the indicators [30]. An AVE value ≥0.5 was used to judge a good convergent validity as this would explain more than half of the variance of its items [28, 30]. To achieve a good convergent validity, an indicator’s factor loading should be >0.7 [30]; however, an indicator’s factor loadings ≥0.4 is still acceptable for exploratory research as proposed previously [32] and was therefore adopted in this study. In addition, divergent validity or discriminant validity was also assessed to check that a construct was truly distinct from other constructs by empirical standards [30]. This assessment was conducted by comparing cross loadings within constructs and between constructs. We used two criteria to assess divergent validity of the construct. The Fornell and Larcker [31] criterion checks if the square root of AVE for each construct is larger than the correlation estimate of the other factor. The Heterotrait-Monotrait (HTMT) score (ranging between -1 to 1) was also calculated, and scores less than 0.85 were used to indicate the two constructs are different [33]. To measure collinearity of the constructs, we used variance inflation factor (VIF), and values of less than 5 were judged to be evidence of new substantial collinearity [30].

At the second stage, we employed a bootstrapping procedure to evaluate the structural model empirically (with 5000 bootstrap samples and 728 bootstrap cases; using no sign changes) to calculate significant values for all paths [30]. We calculated the coefficient value of determination R^2^ and the path coefficients, including the T-value and *P*-value. The R^2^ criterion value was adopted from previous recommendations: 0.02 as small, 0.13 as medium and 0.26 as large [30]. To evaluate our hypotheses, we considered path coefficients with a T-value >1.96 and a *P*-value <0.05 as significant. All PLS-SEM analyses were performed using SmartPLS 3 software.

## Results

The sampling procedure included a total of 768 persons, of whom 728 responded to the survey (response rate = 94.8%). A majority (54.5%) of participants were male and the mean age of respondents was 40 years (Table 2). The vast majority of respondents were Malay (60.3%), followed by Chinese (23.4%) and Indians (19.9%). Approximately 29.4% participants worked in the private sector, 20.4% were self-employed entrepreneurs and 19.9% were unemployed. Less than 2% of participants had no schooling and the mean monthly income was RM 4421.21 (USD 1084).

**Table 2:** Demographic distribution of 728 study participants in Selangor, Malaysia, 2016.

Variable	Frequency (%)	
Age (mean ±SD) (year)	40 ± 11.0	
Sex		
Male	397	(54.5)
Female	331	(45.5)
Ethnicity		
Malay	439	(60.3)
Chinese	170	(23.4)
Indian	116	(19.9)
Others	3	(0.4)
Occupation		
Private employee	214	(29.4)
Self-employment	175	(24.0)
Civil servant	96	(13.2)
Retired	53	(7.3)
Student	26	(3.6)
Others	19	(2.6)
Unemployed	145	(19.9)
Marital status		
Single	139	(19.1)
Married	574	(78.8)
Widowed	9	(1.2)
Divorced	6	(0.8)
Literacy		
Illiterate (never been to school)	13	(1.7)
Literate	715	(98.3)
Education		
Primary	36	(4.9)
Secondary	342	(47.0)
Diploma	188	(25.8)
Degree	123	(16.9)
Postgraduate	26	(3.6)
Monthly income (mean ±SD) (RM)	4421.21 ±3856	

RM: Malaysian ringgit, SD: Standard deviation

The descriptive statistics for perceptions indicted that respondents were indecisive about the perceived susceptibility of HBV infection (Table 3). They indicated that the benefits of HepB vaccination were high and there were few barriers for them to get the HepB vaccination.

**Table 3:** Perceptions of hepatitis B among 728 study participants in Selangor, Malaysia, 2016.

Construct domain	Strongly disagree n (%)	Disagree n (%)	Somewhat disagree n (%)	Neither agree or disagree n (%)	Somewhat agree n (%)	Agree n (%)	Strongly agree n (%)
Cues to action							
CA1	25 (3.4)	24 (3.3)	35 (4.8)	73 (10.0)	113 (15.5)	163 (22.4)	295 (40.5)
CA2	41 (5.6)	52 (7.1)	64 (8.8)	98 (13.5)	100 (13.7)	154 (21.2)	219 (30.1)
CA3	18 (2.5)	29 (4.0)	32 (4.4)	54 (7.4)	110 (15.1)	145 (19.9)	340 (46.7)
Perceived barriers							
PB1	206 (28.3)	190 (26.1)	104 (14.3)	114 (15.6)	60 (8.2)	28 (3.8)	26 (3.6)
PB2	286 (39.2)	133(18.2)	82 (11.2)	100 (13.7)	41 (5.6)	39 (5.3)	47 (6.4)
PB3	250 (34.3)	145 (19.9)	79 (10.8)	87 (11.9)	70 (9.6)	49 (6.7)	48 (6.6)
Perceived benefit							
PBE1	15 (2.1)	11 (1.5)	20 (2.7)	38 (5.2)	124 (17.0)	205 (28.2)	315 (43.3)
PBE2	14 (1.9)	15 (2.1)	27 (3.7)	49 (6.7)	132 (18.1)	233 (32.0)	258 (35.4)
PBE3	18 (2.5)	13 (1.8)	22 (3)	81 (11.1)	153 (21)	199 (27.3)	242 (33.2)
PBE4	23 (3.2)	27 (3.7)	27 (3.7)	66 (9.1)	125 (17.2)	216 (29.7)	244 (33.5)
PBE5	13 (1.8)	14 (1.9)	28 (3.8)	41 (5.6)	125 (17.2)	219 (30.1)	288 (39.6)
Perceived severity							
PSE1	16 (2.2)	25 (3.4)	48 (6.6)	45 (6.2)	93 (12.8)	144 (19.8)	357 (49.0)
PSE2	28 (3.8)	38 (5.2)	48 (6.6)	52 (7.1)	91 (12.5)	173 (23.7)	298 (40.9)
PSE3	18 (2.5)	16 (2.2)	46 (6.3)	65 (8.9)	89 (12.2)	138 (18.9)	356 (48.8)
PSE4	29 (4.0)	54 (7.4)	47 (6.4)	71 (9.7)	115 (15.8)	153 (21.0)	259 (35.5)
Perceived susceptibility							
PSU1	79 (10.8)	92 (12.6)	95 (13)	164 (22.5)	98 (13.4)	126 (17.3)	74 (10.2)
PSU2	88 (12.1)	94 (12.9)	97 (13.3)	158 (21.7)	106 (14.5)	116 (15.9)	69 (9.5)
PSU3	152 (20.9)	123 (16.9)	99 (13.6)	75 (10.3)	105 (14.4)	82 (11.2)	92 (12.6)

Our data indicated that reliability varied across constructs (Table 4). Crohnbach’s α ranged from 0.527 (for perceived barriers) to 0.843 (for perceived benefit) and was above 0.7 (acceptable) for perceived severity and perceived benefit. Composite reliability was above 0.6 (acceptable) for all constructs, and ranged from 0.757 (for perceived barriers) to 0.886 (for perceived benefit).

**Table 4:** Reliability and validity of items within constructs of perceptions among 728 participants in Selangor, Malaysia, 2016.

Construct domain	Indicator	Loadings	Cronbach’s α	Composite Reliability	Average Variance Extracted (AVE)
Cues to action	CA1	0.746	0.685	0.820	0.604
	CA2	0.746			
	CA3	0.837			
Perceived barriers	PB1	0.640	0.527	0.757	0.513
	PB2	0.675			
	PB3	0.820			
Perceived benefit	PBE1	0.767	0.843	0.886	0.609
	PBE2	0.735			
	PBE3	0.798			
	PBE4	0.775			
	PBE5	0.822			
Perceived susceptibility	PSU1	0.553	0.597	0.774	0.540
	PSU2	0.789			
	PSU3	0.832			
Perceived severity	PSE1	0.681	0.795	0.855	0.599
	PSE2	0.906			
	PSE3	0.699			
	PSE4	0.789			

Convergent validity of items within construct was tested with two measures (AVE and outer factor loadings). Our data indicated that AVE exceeded 0.5 for all constructs (Table 4). This indicates each construct explains over 50% of the variance of its items. The values of the outer factor loadings ranged from 0.553 to 0.906 showing that all instances were above 0.70 except for two items in perceived barriers, one item in perceived susceptibility and two items in perceived severity (Table 5).

**Table 5:** Outer factor loadings analysis of construct items among 728 participants in Selangor, Malaysia, 2016.

	Cues to action	Perceived barriers	Perceived benefit	Perceived susceptibility	Perceived severity	Willingness to pay
CA1	**0.746**	-0.101	0.348	0.099	0.406	0.111
CA2	**0.746**	-0.101	0.269	0.084	0.324	0.108
CA3	**0.837**	-0.093	0.329	-0.001	0.329	0.173
PB1	-0.013	**0.640**	-0.135	0.101	-0.047	0.097
PB2	-0.117	**0.675**	-0.176	0.169	-0.046	0.039
PB3	-0.121	**0.820**	-0.236	0.058	-0.031	0.064
PBE1	0.349	-0.173	**0.767**	0.012	0.353	0.045
PBE2	0.351	-0.130	**0.735**	0.042	0.349	0.027
PBE3	0.309	-0.183	**0.798**	0.045	0.300	0.094
PBE4	0.262	-0.241	**0.775**	0.077	0.260	0.060
PBE5	0.337	-0.250	**0.822**	0.068	0.373	0.040
PSU1	0.060	0.058	0.070	**0.553**	0.119	0.088
PSU2	0.064	0.136	0.036	**0.789**	0.029	0.175
PSU3	0.035	0.109	0.055	**0.832**	0.002	0.224
PSE1	0.478	-0.150	0.398	0.033	**0.681**	0.027
PSE2	0.367	-0.065	0.342	0.079	**0.906**	0.099
PSE3	0.408	-0.061	0.337	0.033	**0.699**	0.038
PSE4	0.273	0.040	0.296	-0.031	**0.789**	0.063
WTP	0.175	0.090	0.070	0.238	0.086	**1.000**

To assess discriminant validity, we compared cross loadings within constructs and between constructs and our data verified that the different items loaded onto their specific construct consistently (Table 5). Analyses of discriminant validity using Fornell and Larcker and HTMT criterion are provided in Table 6. Using the Fornell and Larcker criterion, the square root of AVE of each construct was larger than the correlation estimate of the factors. Discriminant validity according to the HTMT criterion revealed that the highest value, 0.538, was between cues to action and perceived benefit (Table 6).

**Table 6:** Fornell-Larcker and Heterotrait-Monotrait criterion for discriminant validity in a study of perceptions of hepatitis B among 728 participants in Selangor, Malaysia, 2016.

Fornell and Larcker criterion	CA	PB	PBE	PSE	PSU	WTP
Cues to action	**0.777**					
Perceived barriers	-0.124	**0.716**				
Perceived benefit	0.404	-0.261	**0.780**			
Perceived severity	0.065	0.143	0.067	**0.735**		
Perceived susceptibility	0.445	-0.056	0.413	0.043	**0.774**	
Willingness to pay	0.175	0.090	0.070	0.238	0.086	**1.000**
Heterotrait-Monotrait criterion	CA	PB	PBE	PSE	PSU	WTP
Cues to action	-					
Perceived barriers	0.199					
Perceived benefit	0.538	0.365				
Perceived severity	0.140	0.258	0.099			
Perceived susceptibility	0.662	0.154	0.537	0.121		
Willingness to pay	0.201	0.128	0.074	0.283	0.081	

Square roots of AVE have shown diagonally (in bold)

Relative to the dependent variable (WTP), the collinearity analysis between constructs found that the variance inflation factor values for all constructs were less than 5 (cues to action (1.350), perceived barriers (1.111), perceived benefit (1.391), perceived severity (1.035), and perceived susceptibility (1.363). These findings indicate no substantial evidence of multicollinearity.

Four out of the six hypothesised relationships were significant: cues to action, perceived susceptibility, and perceived barriers were significantly associated with WTP, and the construct of perceived barriers was significantly associated with perceived benefit (Table 7).

**Table 7:** Path coefficient assessment in a study of perceptions of hepatitis B among 728 participants in Selangor, Malaysia, 2016.

Hypothesis	Path coefficient	95% CI		T-Value	*P*-value
		Lower	upper		
Cues to action → WTP	0.166	0.084	0.246	3.914	<0.001
Perceived susceptibility → WTP	0.214	0.146	0.282	6.032	<0.001
Perceived severity → WTP	0.005	-0.074	0.116	0.098	0.922
Perceived barriers →WTP	0.082	-0.004	0.154	2.096	0.036
Perceived benefit → WTP	0.008	-0.097	0.072	0.188	0.851
Perceived barriers → Perceived benefit	-0.261	-0.373	-0.221	6.578	<0.001

**Fig 3:**
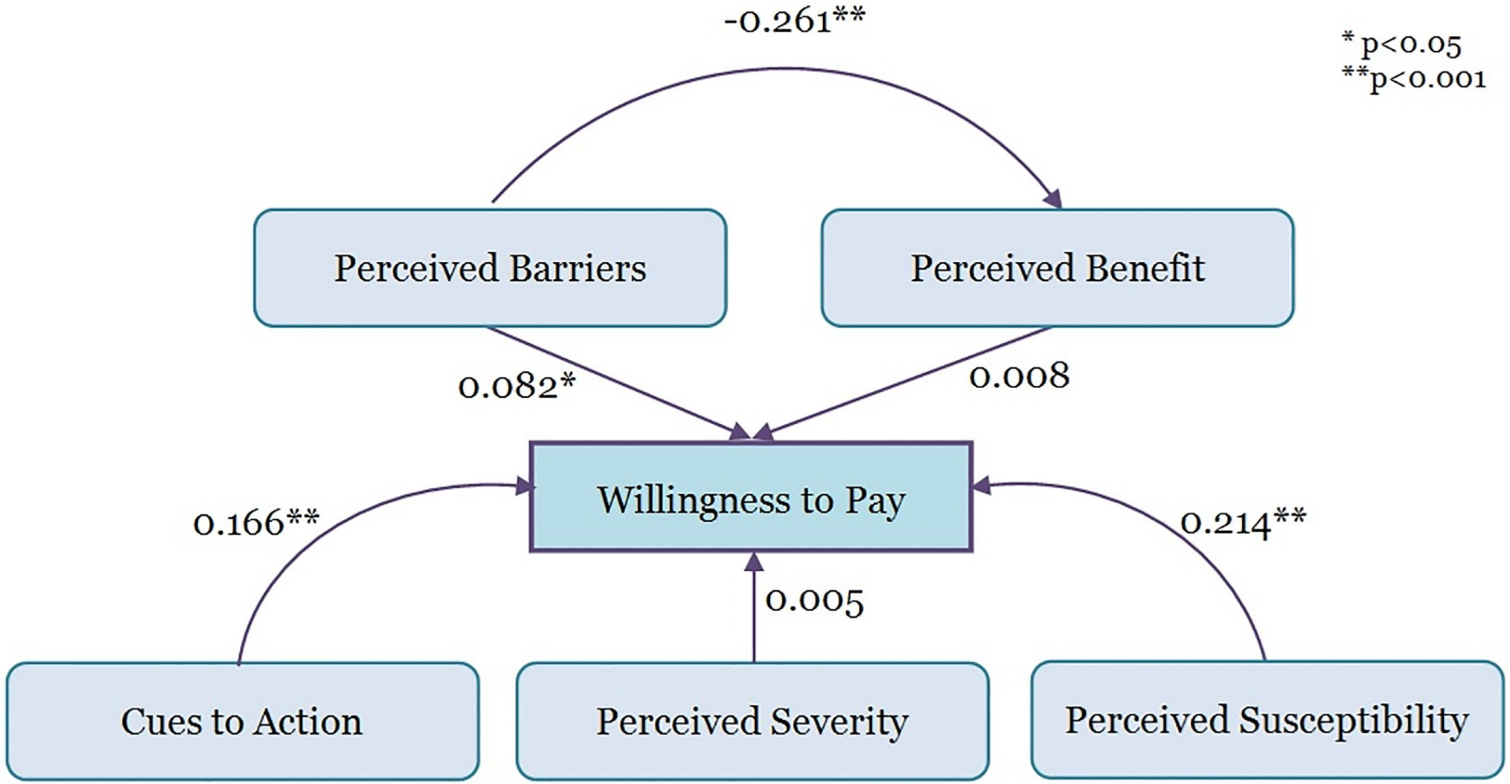
Structural equation model of willingness to pay for hepatitis B among 728 participants in Selangor, Malaysia, 2016.

Fig 3 graphically depicts the relationship between the different constructs and WTP. There was a significant, positive relationship between three constructs (perceived barriers, perceived susceptibility, and cues to action) and WTP, and this model all together accounted for 8.8% of the variation in WTP. There was also a significant, negative relationship between perceived barriers and perceived benefit, which accounted for 6.8% of the variation in perceived benefit.

## Discussion

Chronic HBV infection can lead to highly morbid conditions and death. A robust pediatric immunization program in Malaysia will likely lead to a decrease in HBV transmission and reduce the number of chronic HepB carriers, but the lack of publicly funded adult vaccinations may impede efforts to eliminate HepB in the country. In the absence of a public immunization program for adults, it is important to identify which factors impact individuals’ vaccine decision-making. Using a PLS-SEM, this study found that there was a significant, positive relationship between three constructs (perceived barriers, perceived susceptibility, and cues to action) and WTP, and there was a significant, negative relationship between perceived barriers and perceived benefit.

### Health behaviour model constructs in previous literature

Perceived susceptibility and cues to action have both been previously related to a variety of health care and healthy behaviours. Perceived susceptibility is a dominant perception analysed by many previous researchers [20, 21, 34–36]. In this research, perceived susceptibility had a positive relationship with WTP, with a greater strength of association than any other construct. This implies that the more that respondents perceived susceptibility, the more they were willing to pay for the HepB vaccine. This relationship is similar to other studies, for instance, those related to breast cancer treatments [37] and genetic testing for cancer risk [38]. Interestingly, the descriptive statistics of items within perceived susceptibility indicate that many respondents were indecisive about the possibility of the HBV infection and unsure about their body’s capability to fight HBV infection. However, a large proportion expressed worry that they will become infected with HBV. These findings are possible points to consider when promoting the vaccine or educating the public.

Cues to action had a positive relationship with WTP for the HepB vaccination, which implies that when respondents had a greater internal incentive for living healthy without HepB, they were more willing to pay for HepB vaccination. Cues to action are a common feature of many health behaviour models, although some studies have not found a relationship between cues to action and vaccination behaviour; for example, in one study among young women in Australia, cues to action did not predict human papillomavirus vaccination intention or vaccine behavior [39].

We hypothesized that there would be a negative relationship between perceived barriers and WTP. This direction has been observed in WTP for genetic testing for cancer risk [38]. However, in this study, the opposite association was observed: higher barriers was associated with greater WTP. Notably, few respondents expressed strong agreement with the barriers, which limits our ability to model what happens when perceived barriers are high. Theoretically, when the barrier is higher, there is a lower chance of adopting a new behaviour [40]. Since in this study respondents perceived few barriers to adopt a new behaviour (vaccinating themselves against HBV), the probability of getting vaccinating (or being willing to pay for vaccination) was correspondingly higher.

This study proposed a new path in the Health Belief Model between perceived barriers and perceived benefit. Although this direct relationship was never examined previously, this relationship is similar to a previous study, which proposed a relationship between perceived barriers and vaccine acceptance [19]. Bodenheim et al [19] reported that vaccine acceptance was determined by beliefs in vaccine safety and efficacy. Another study also highlighted that sustained vaccine coverage can only be achieved if public confidence and trust in the vaccine are high [41]. In summary, respondents’ perceptions of the barriers to HepB vaccination should be identified and overcome to increase the perception of benefits.

Perceived benefit and perceived severity were hypothesized to have a positive relationship with WTP for HepB vaccination–and this type of relationship has been identified in a previous study [38], but no significant relationship for these constructs was found in this study. This may be due to respondents’ relatively high perceived benefit and perceived severity, which limits the space to test for the impact of variation in the construct on the outcome. Moreover, perceived benefit, although not linked directly to WTP, was itself an important component of our model, being significantly linked to perceived barriers. Future research which looks at actual vaccination behaviours can clarify the linkages among these different constructs.

### Policy and program recommendations

The findings from this study suggest several items of action that the government and hospitals can undertake to increase HepB vaccine uptake among adults. The government (e.g., the Ministry of Health) could increase the nation’s awareness of HepB vaccination through social communications. For example, short audio or video clips could be presented to the visitors who are waiting in cue for services at public hospitals. Government clinics or hospitals could increase vaccination services and HepB screening especially during weekends to accommodate those who have a busy working schedule. These actions could decrease barriers (leading to greater perceived benefits), and modify individual’s perceptions of susceptibility and cues to action, leading to greater WTP. Additionally, private clinics and hospitals could encourage free HepB screening to their patients by giving a discount for HepB vaccination if the patient completes the screening. This type of program could facilitate a good relationship with patient-customers, raise awareness of HepB, and increase vaccination uptake.

### Strengths and limitations

Several limitations are possible in this study. Participants could have given more expected answers to the interviewer, leading to a social desirability bias. Social desirability bias could presumably lead to more individuals saying that they would be willing to pay for the vaccine, which could dilute the associations between the constructs of interest and WTP. However, we do not have empirical evidence on how much social desirability could affect responses to the WTP question or to other items on the survey. The sampling of this study was a strength, in that we attempted a random selection of individuals from Selangor state through community-based samples.

## Conclusions

This study highlights the importance of perceptions in adults’ WTP for hepatitis B vaccine. Increasing vaccination coverage is essential within Malaysia, which has a high burden of HepB disease, and which faces the WHO goal of eliminating HepB by 2030. This analysis shows that cues to action, perceived susceptibility and perceived barriers have a direct influence on WTP, and that perceived barriers impacted perceived benefits. Policies and programs should be developed that can modify individuals’ thoughts about disease risk, their obstacles to obtaining a preventive action, and their readiness to obtain a vaccine. Such programs include educational materials about disease risk and clinic visits that pair HepB screening and vaccination.

## Supporting information

S1 Supporting Information (PDF)
