# Supplementary material for: The relationship between perceptions and self-paid hepatitis B vaccination: A structural equation modeling approach
Source: PLoS One. 2018 Dec 6;13(12):e0208402. doi: 10.1371/journal.pone.0208402 (PMC6283584; doi:10.1371/journal.pone.0208402)
Supplement: S1 Supporting Information — (PDF) [file pone.0208402.s001.pdf]

Additional file 1 - Questionnaire used in the study

Demographic data

1. Age: ______ Years

2. Gender
- ☐ Male
- ☐ Female

3. Race
- ☐ Malay
- ☐ Chinese
- ☐ Indian
- ☐ Others: ________________________

4. Marital status
- ☐ Single
- ☐ Married
- ☐ Divorced
- ☐ Widow/Widower

5. Occupation
- ☐ Public sector
- ☐ Private sector
- ☐ S Self-employed
- ☐ Student
- ☐ Retired
- ☐ Unemployment
- ☐ Others: ________________________

6. Educational attainment
- ☐ Never been to school
- ☐ Primary school
- ☐ Secondary school
- ☐ Diploma/certificate
- ☐ Undergraduate
- ☐ Post graduate

7. Household monthly income: ______ RM

State the extent to which you agree with each of the following. Tick(√) your answer.

Willingness to Pay

1- Strongly disagree
2- Disagree
3- Somewhat disagree
4- Neither agree or disagree
5- Somewhat agree
6- Agree
7- Strongly agree

No	Question							
1	I agree to pay for hepatitis B vaccine at the expense of my own	1	2	3	4	5	6	7

Perceived Susceptibility

1- Strongly disagree
2- Disagree
3- Somewhat disagree
4- Neither agree or disagree
5- Somewhat agree
6- Agree
7- Strongly agree

No	Question							
1	I am less likely than most people to get infected with hepatitis B virus	1	2	3	4	5	6	7
2	My body could fight off hepatitis B virus infection.	1	2	3	4	5	6	7
3	I never worry about getting infected with hepatitis B virus	1	2	3	4	5	6	7

Perceived Severity

1- Strongly disagree
2- Disagree
3- Somewhat disagree
4- Neither agree or disagree
5- Somewhat agree
6- Agree
7- Strongly agree

No	Question							
1	I believe that I am at a higher risk of hepatitis B virus infection	1	2	3	4	5	6	7
2	I believe that my ethnic group is at a higher risk of hepatitis B virus infection	1	2	3	4	5	6	7
3	I belief that hepatitis B virus infection is a serious disease	1	2	3	4	5	6	7
4	I believe that HB infection leads to death	1	2	3	4	5	6	7

Perceived Benefit

1- Strongly disagree
2- Disagree
3- Somewhat disagree
4- Neither agree or disagree
5- Somewhat agree
6- Agree
7- Strongly agree

No	Question							
1	I believe if I get the hepatitis B vaccine, I shall be protected from hepatitis B virus infection	1	2	3	4	5	6	7
2	If I take the hepatitis B vaccine, it will reduce my worry about liver disease	1	2	3	4	5	6	7
3	I believe in the effectiveness of the hepatitis B vaccine now	1	2	3	4	5	6	7
4	I believe a vaccine for hepatitis B strengthens the immune system against hepatitis B virus	1	2	3	4	5	6	7
5	I believe that getting the hepatitis B vaccine is a good way to protect yourself from hepatitis B virus infection	1	2	3	4	5	6	7

Perceived Barriers

1- Strongly disagree
2- Disagree
3- Somewhat disagree
4- Neither agree or disagree
5- Somewhat agree
6- Agree
7- Strongly agree

No	Question							
1	I believe that the vaccination is not effective for me	1	2	3	4	5	6	7
2	I believe that the hepatitis B vaccination is likely to cause more harm than good	1	2	3	4	5	6	7
3	I do not have the time to get the vaccination	1	2	3	4	5	6	7

Cues to action

1- Strongly disagree
2- Disagree
3- Somewhat disagree
4- Neither agree or disagree
5- Somewhat agree
6- Agree
7- Strongly agree

No	Question							
1	I think the screening for HB infection is a good practice	1	2	3	4	5	6	7
2	An additional dose (booster) of the vaccine for hepatitis B should be taken when needed	1	2	3	4	5	6	7
3	I think all members of the family and friends should get the hepatitis B vaccine	1	2	3	4	5	6	7

Data demografis

1. Umur: ☐ Tahun

2. Jantina
   - ☐ Lelaki
   - ☐ Perempuan

3. Keturunan
   - ☐ Malayu
   - ☐ Cina
   - ☐ India
   - ☐ Lain-lain: ________________________

4. Taraf perkahwinan:
   - ☐ Bujang
   - ☐ Kahwin
   - ☐ Bercerai
   - ☐ Janda/Duda

5. Occupation
   - ☐ Sektor awam
   - ☐ Sektor swasta
   - ☐ Bekerja sendiri
   - ☐ Pelajar
   - ☐ Bersara
   - ☐ Tidak bekerja
   - ☐ Lain-lain: ________________________

6. Pendidikan
   - ☐ Tidak pernah kesekolah
   - ☐ Sekolah Rendah
   - ☐ Sekolah Menengah
   - ☐ Diploma/Sijil
   - ☐ Sarjana Muda
   - ☐ Pascasiswazah

7. Berapakah pendapatan bulanan isirumah Anda: ☐ RM

Nyatakan sejauh mana anda bersetuju dengan setiap yang berikut.
Tandakan (√) jawapan anda.

Willingness to Pay

1- Sangat tidak setuju
2- Tidak bersetuju
3- Agak tidak setuju
4- Antara setuju dan tidak bersetuju
5- Agak setuju
6- Setuju
7- Sangat bersetuju

No	Question							
1	Saya setuju untuk membayar vaksin hepatitis B atas perbelanjaan saya sendiri	1	2	3	4	5	6	7

Perceived Susceptibility

2- Sangat tidak setuju
2- Tidak bersetuju
3- Agak tidak setuju
4- Antara setuju dan tidak bersetuju
5- Agak setuju
6- Setuju
7- Sangat bersetuju

No	Soalan							
1	Peluang saya untuk dijangkiti dengan HepB adalah sangat sedikit jika dibandingkan dengan orang lain.	1	2	3	4	5	6	7
2	Badan saya boleh melawan jangkitan HepB.	1	2	3	4	5	6	7
3	Saya tidak pernah bimbang akan dijangkiti oleh HepB	1	2	3	4	5	6	7

Perceived Severity

1- Sangat tidak setuju
2- Tidak bersetuju
3- Agak tidak setuju
4- Antara setuju dan tidak bersetuju
5- Agak setuju
6- Setuju
7- Sangat bersetuju

No	Soalan							
1	Saya percaya bahawa saya berisiko tinggi untuk mendapat jangkitan HepB.	1	2	3	4	5	6	7
2	Saya percaya bahawa keturunan saya berisiko tinggi untuk jangkitan HepB.	1	2	3	4	5	6	7
3	Saya percaya bahawa jangkitan HepB adalah penyakit yang serius.	1	2	3	4	5	6	7
4	Saya percaya bahawa jangkitan HepB membawa kepada kematian.	1	2	3	4	5	6	7

Perceived Benefit

1- Sangat tidak setuju
2- Tidak bersetuju
3- Agak tidak setuju
4- Antara setuju dan tidak bersetuju
5- Agak setuju
6- Setuju
7- Sangat bersetuju

No	Soalan							
1	Saya percaya jika saya mendapat vaksin HepB, saya boleh dilindungi dari jangkitan HepB.	1	2	3	4	5	6	7
2	Jika saya mengambil vaksin HepB ia akan megurangkan kebimbangan saya mengenai penyakit hati	1	2	3	4	5	6	7
3	Saya percaya terhadap keberkesanan vaksin HepB sekarang.	1	2	3	4	5	6	7
4	Saya percaya vaksin HepB menguatkan system imun saya terhadap virus HepB.	1	2	3	4	5	6	7
5	Saya percaya bahawa mendapat vaksin HepB adalah cara yang baik untuk melindungi diri daripada jangkitan virus HepB	1	2	3	4	5	6	7

Perceived Barriers

1- Sangat tidak setuju
2- Tidak bersetuju
3- Agak tidak setuju
4- Antara setuju dan tidak bersetuju
5- Agak setuju
6- Setuju
7- Sangat bersetuju

No	Soalan							
1	Saya percaya bahawa vaksinasi tidak berkesan untuk saya.	1	2	3	4	5	6	7
2	Saya percaya bahawa vaksinasi HepB mendatangkan lebih banyak keburukan daripada kebaikan.	1	2	3	4	5	6	7
3	Saya tidak mempunyai masa untuk mendapatkan vaksinasi.	1	2	3	4	5	6	7

Cues to action

1- Sangat tidak setuju
2- Tidak bersetuju
3- Agak tidak setuju
4- Antara setuju dan tidak bersetuju
5- Agak setuju
6- Setuju
7- Sangat bersetuju

No	Soalan							
1	Saya rasa saringan untuk jangkitan HepB adalah amalan baik.	1	2	3	4	5	6	7
2	Dos tambahan vaksin HepB perlu diambil apabila diperlukan.	1	2	3	4	5	6	7
3	Saya rasa semua ahli keluarga dan rakan-rakan saya perlu mendapatkan vaksin HepB.	1	2	3	4	5	6	7
